# Effects of Fasting-Mimicking Diet and Specific Meal Replacement Foods on Blood Glucose Control in Patients with Type 2 Diabetes: A Randomized Controlled Trial

**DOI:** 10.1155/2020/6615295

**Published:** 2020-12-05

**Authors:** Fang Tang, Xuan Lin

**Affiliations:** ^1^Wuhan University of Science and Technology School of Medicine, Wuhan, 430081 Hubei, China; ^2^Department of Endocrinology, CR & WISCO General Hospital Affiliated to Wuhan University of Science and Technology, Wuhan, 430080 Hubei, China

## Abstract

Type 2 diabetes represents a serious societal health problem due to the vulnerability to cardiovascular events. Diet therapy is the most basic treatment for type 2 diabetes. The present study was conducted to study the effect of a fasting-mimicking diet and specific meal replacement foods on blood glucose control in patients with type 2 diabetes. Our study included 100 patients with type 2 diabetes who underwent a physical examination which were enrolled and randomly assigned as 50 patients each to the test group (with low energy-specific meal replacement meals during a fasting-mimicking diet) and the control group (with specific meal replacement foods given normal adult doses). After 4 months, efficacy indicators which were fasting blood glucose, 2-hour postprandial venous blood glucose, and glycosylated haemoglobin of the experimental group were all lower than those of the control group (*P* < 0.05); observation indicators that include body mass index, waist circumference, blood lipids (triglyceride, cholesterol, and low-density lipoprotein), and blood pressure levels were all lower than the control group, and high-density lipoprotein levels were all higher than the control group (all *P* < 0.05). Both groups of fasting blood glucose, 2-hour postprandial venous blood glucose, and blood pressure had a relatively stable downward trend, but the experimental group had a more significant decline. In conclusion, the study revealed that a fasting-mimicking diet and specific meal replacement foods can safely and effectively reduce weight and improve metabolic syndrome in patients with type 2 diabetes.

## 1. Introduction

The latest surveys of obesity and metabolic syndrome show that the prevalence of type 2 diabetes in overweight (body mass index (BMI) 25–27.5 kg/m^2^) and obese (BMI > 27.5 kg/m^2^) populations in China is 12.8% and 18.5%, respectively. The global prevalence of type 2 diabetes is increasing annually, and the latest data from the International Diabetes Federation (IDF) 2019 showed that approximately 463 million adults worldwide have diabetes. It is estimated that, by 2030 and 2045, the number of individuals with diabetes will reach 578.4 and 700.2 million, respectively. Therefore, the prevention and treatment of type 2 diabetes are particularly important. Diet is presently considered a key factor in the management of patients with obese type 2 diabetes. The fasting-mimicking diet (FMD) involves alternate fasting and consumption of a calorie-restricted diet wherein only 25% of the normal intake is consumed on fasting days and normal caloric intake is maintained on eating days [1]. A large number of studies have shown that FMD is an emerging dietary intervention that can effectively reduce and control weight in obese patients with type 2 diabetes, improve impaired glucose regulation, and improve the risk factor level of diabetes [2–4]. A study by Cheng et al. at the University of Southern California found that FMD can promote the renewal and growth of pancreatic cells and promote insulin production. Moreover, FMD can reverse the symptoms of type 1 and type 2 diabetes in mouse models and is effective in insulin in vitro in patients with type 1 diabetes [5]. Furthermore, Stekovic et al. of Graz University of Technology in Austria evaluated the FMD in healthy, nonobese individuals and found that FMD could reduce the body weight, improve the ratio of fat to lean meat, decrease blood lipids, and reduce the levels of the cardiovascular and inflammatory markers related to aging [6]. Several studies have reported that FMD can improve the health of model organisms and prolong their life expectancy, with good physiological regulatory significance in overweight individuals with or without type 2 diabetes as well as in healthy and nonobese participants. However, the determination of 25% of energy intake on a fasting day has not been previously addressed.

To fill this gap, this study developed the Food for Special Medical Purpose (FSMP) protocol, which was quantified and applied to fasting days. The FSMP is a specially formulated food that is developed to meet the special needs of a nutrient or diet for people with restricted eating, digestive and absorption disorders, metabolic disorders, or specific disease states. This study analyzed the effect of FMD combined with specific substitute foods on blood glucose in patients with type 2 diabetes mellitus.

## 2. Materials and Methods

### 2.1. Ethics Approval and Consent to Participate

This study conforms to the principles of the Declaration of Helsinki and was conducted with the approval of the Ethics Committee of CR & WISCO General Hospital Affiliated to Wuhan University of Science and Technology and the Chinese Clinical Trial Registry (grant no. ChiCTR2000032968). It was registered on 17 May and 2020-retrospectively registered, http://www.chictr.org.cn/ChiCTR2000032968. Permissions were obtained from all the relevant authorities of the hospital before enrollment of the patients, and written informed consent was sought from all the participants before enrollment. Necessary measures were taken to maintain confidentiality of the data and privacy of the participants.

### 2.2. General Information

From January 2018 to December 2018, 100 patients with type 2 diabetes and BMI ≥ 28 kg/m^2^ who underwent a physical examination at the endocrine clinic and physical examination centre of CR & WISCO General Hospital Affiliated to Wuhan University of Science and Technology were selected for a 4-month simulated fasting diet. The random number table method was used to randomly assign 50 patients each to the test and control groups.

### 2.3. Inclusion and Exclusion Criteria

#### 2.3.1. Inclusion Criteria

The inclusion criteria are as follows: meeting the World Health Organization (WHO) diagnostic criteria for type 2 diabetes, adult male and female participants (age 18–65 years, both inclusive), BMI ≥ 28 kg/m^2^, good glycemic control (glycosylated haemoglobin (HbA1c) 7.0–10.0%, both values inclusive), relatively stable weight (weight change ≤ 10% for at least 3 months before study inclusion), and willingness to use a glucometer. The blood glucose was measured and recorded in the participant's diary.

#### 2.3.2. Exclusion Criteria

The exclusion criteria are as follows: participation in other clinical trials within 3 months preceding study enrolment; systolic blood pressure ≥ 180 mmHg and/or diastolic blood pressure ≥ 110 mmHg during screening visits; regular use of insulin, oral steroids, or anti-inflammatory drugs; diagnosis of cardiovascular disease; stroke, gastrointestinal disease, chronic nephritis, hepatobiliary disease, or renovascular disease; pregnant and lactating women; relatives of the investigators of the trial, employees of the hospital, or others who were related to the trial personnel; a major illness or physical weakness; and the investigator's judgement that the participant may be unable to complete this study.

### 2.4. Test Design

The FMD meal replacement food used in the trial was developed by the partner unit Maide Technology Company Limited by Shares (Maide Technology Co., Ltd., Wuhan, China) and had good safety. The meal replacement formula comprised avocado, oatmeal, green food nutrition powder, salt, and bitter melon powder. According to the book *Calories and Protein Intake* published by the WHO, a healthy woman or man needs 1800–1900 and 1980–2340 Kcal per day. This 4-month study included meal replacement intervention for 3 months and normal diet for the last month. The test group consumed FMD meal replacement powder from Monday to Friday in the second week of a month and ate normally for the rest of the month. The energy provision on the first day and the second to fifth days was 1196 and 805 Kcal, respectively (formula for patients weighing 75 kg). The investigator adjusted the meal replacement amount of the patient according to the weight and physical consumption, whereas the control group consumed meal replacement powder from Monday to Friday of the second week of the month (the same composition and different caloric composition as those in the test group; the calories met the abovementioned recommended daily requirement for normal adults) and ate normally for the remainder of the month.

During the test period, all patients received metformin hydrochloride tablets (Gehuazhi, Sino-American Shanghai Bristol-Myers Squibb Pharmaceutical Co., Ltd., Shanghai, China), 0.5 g, once daily as treatment for diabetes, and patient compliance was ascertained. For patients in the test group who had hypoglycemia or other discomforts on FMD meal replacement days, the metformin hydrochloride dosage was adjusted or discontinued after assessment by the endocrinologist.

### 2.5. Test Indices

The efficacy indicators were fasting plasma glucose (FPG), 2-hour postprandial glucose (2hPG), and HbA1c. The observation indicators included BMI, waist circumference, blood lipids (triglyceride (TG), cholesterol, high-density lipoprotein (HDL), and low-density lipoprotein (LDL)), and blood pressure. The FPG, 2hPG, and blood pressure measurements were obtained every week to evaluate the fluctuation of indicators in the study cycle because of the short-term differences. The remaining indicators are checked before and after the test.

### 2.6. Statistical Analysis

All data were analyzed in SPSS 21.0 statistical software. The present report is based on the intention-to-treat analysis. Normal distribution of continuous variables was assessed by the Kolmogorov–Smirnov test. Results are expressed as mean ± standard deviation (SD). Data with normal distribution are expressed as (*χ* ± *s*). Intergroup comparisons were analyzed by the *t*-test. The observation index volatility was measured by repeated measures analysis of variance. Numerical data was expressed as percentages and analyzed with the chi-square test. Differences were statistically significant at *P* < 0.05.

## 3. Results

**Figure 1:**
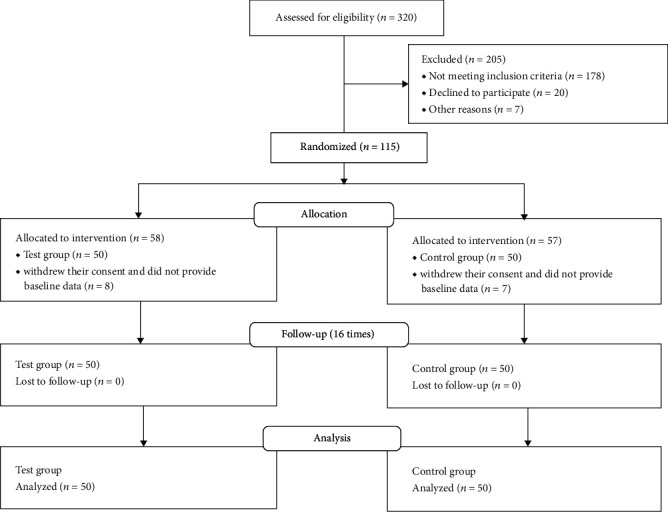
CONSORT diagram showing the number of participants at enrollment and follow-ups.

We recruited 320 type 2 diabetes patients and selected 115/320 (35.9%) of them to enter this study based on the inclusion and exclusion criteria. Out of 115 participants, 58 (50.4%) were randomized into the test group, and 57 (49.6%) were randomized into the control group. Only 50/58 (86.2%) of the test group received meal replacement diet intervention and also provided baseline data in the test group arm, and only 50/57 (87.7%) of the control group received adult-recommended calorie meal replacement diets and provided baseline data in the control group arm. There were *n* = 8 and *n* = 7 patients in the test group and the control group arm, respectively, who withdrew their consent after randomization and did not provide baseline data. There was no loss to follow-up in the 16-time visits (see Figure 1).

### 3.1. Study Participant Characteristics before Experimental Intervention

A total of 100 patients with type 2 diabetes were included in this study, the general information of the two groups of patients was compared, and the difference was not statistically significant (all *P* > 0.05; see Table 1).

### 3.2. Data Analysis of Efficacy Indicators after Experimental Intervention

Table 2 shows the values after 4 months of experimental intervention. The FPG was 5.25 ± 0.23 mmol/L in the experimental group and 6.27 ± 0.37 mmol/L in the control group, the 2hPG was 7.02 ± 2.27 mmol/L and 8.33 ± 0.89 mmol/L, respectively, and the HbA1c was 6.47 ± 0.51% and 7.50 ± 0.50%, respectively. All glycemic parameters showed significant statistical intergroup differences (*P* < 0.05; see Table 2).

### 3.3. Observation Index Data Analysis after Experimental Intervention

After 4 months of experimental intervention, the BMI of the experimental and control groups were 25.04 ± 1.00 and 28.99 ± 0.99 kg/m^2^, respectively, and the waist circumference was 90.82 ± 4.26 and 98.38 ± 4.27 cm, respectively, showing significant statistical intergroup differences (all *P* < 0.05; see Table 3).

The systolic blood pressure in the experimental and the control group was 141.10 ± 6.67 and 149.08 ± 5.50 mmHg, respectively, and diastolic blood pressure was 80.50 ± 5.97 and 85.20 ± 6.12 mmHg, respectively, showing significant statistical differences (all *P* < 0.05; see Table 4).

The TC of the experimental group and the control group were 3.63 ± 0.97 and 5.60 ± 0.94 mmol/L, respectively. The TG was 2.05 ± 0.54 and 3.51 ± 0.47 mmol/L, respectively. LDL-C was 1.97 ± 0.49 and 3.38 ± 0.62 mmol/L, respectively. HDL-C was 2.28 ± 0.50 and 1.42 ± 0.27 mmol/L, respectively, showing significant statistical intergroup differences (all *P* < 0.05; see Table 5).

### 3.4. Overall Trend Analysis of Relevant Data after Test Intervention

**Figure 2:**
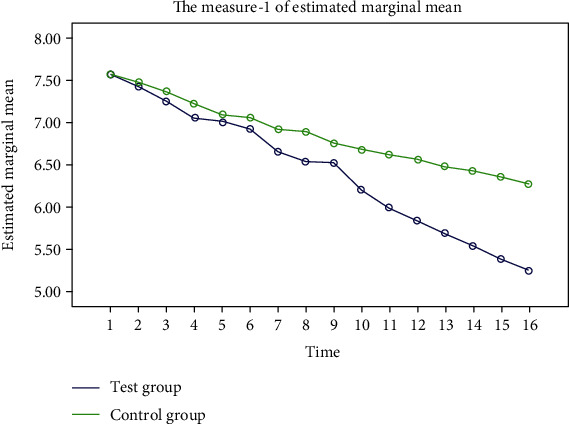
Trend of FPG changes during the two groups of experiments. The blue represents the experimental group, and the green represents the control group. After 16 weeks of the experimental cycle, FPG in both groups showed a steady decline, but the decline was more significant in the experimental group.

**Figure 3:**
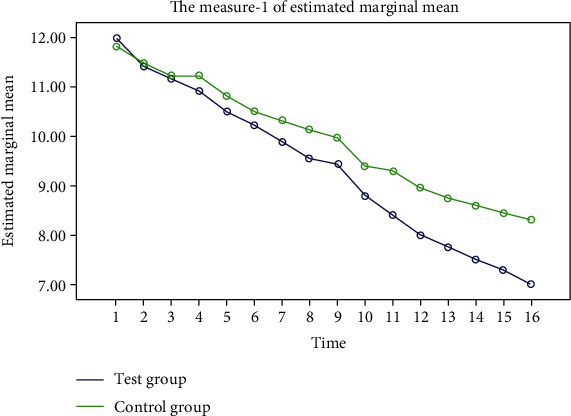
Trend of 2hPG changes during the two groups of experiments. The blue represents the experimental group, and the green represents the control group. After 16 weeks of the experiment cycle, both groups showed a steady decline in 2hPG, but the decline was more significant in the experimental group.

**Figure 4:**
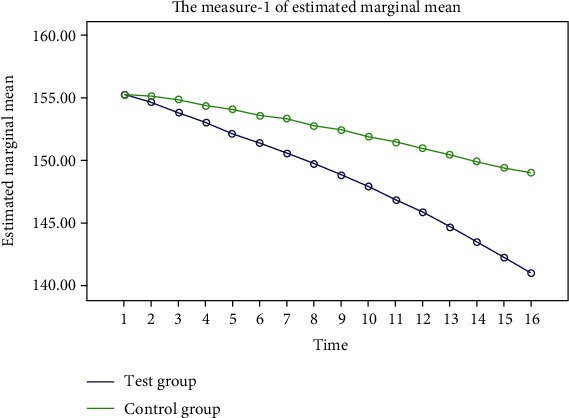
Trend of systolic pressure changes during the two groups of experiments. The blue represents the experimental group, and the green represents the control group. After 16 weeks of the experiment cycle, the systolic pressure in both groups showed a stable trend of decline, but the decline in the experimental group was more significant.

**Figure 5:**
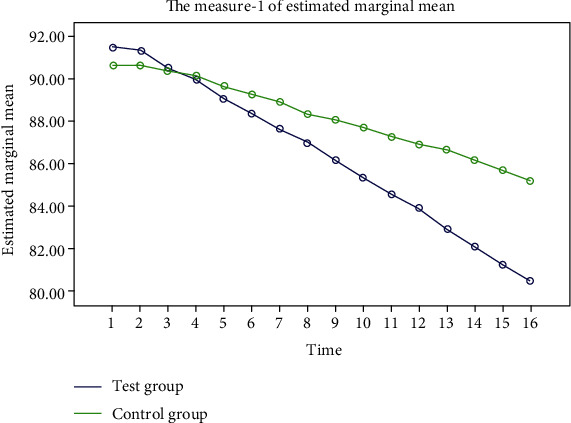
Trend of diastolic pressure changes during the two groups of experiments. The blue represents the experimental group, and the green represents the control group. After 16 weeks of the experiment cycle, the diastolic pressure in both groups showed a stable trend of decline, but the decline in the experimental group was more significant.

The FPG, 2hPG, and blood pressure in the two groups showed a steady decline, and the decline in the test group was more significant. “Some additional figure file shows this in more detail (see Figures 2–5).”

## 4. Discussion

Diet therapy is the most basic therapy for type 2 diabetes. During the treatment of obesity type 2 diabetes, diet therapy is helpful to reduce weight; correct protein, sugar, and lipid metabolism; and improve the metabolic syndrome [7]. As a subclass of diet therapy, FMD has been shown to benefit the reduction of the risks for diabetes and cardiovascular disease and modulate the hormones that regulate hunger and satiety [8]. Whereas all forms of diet therapy exist in patients with long-term adherence to poor compliance, disadvantages such as inaccurate grasp of the value lead to poor dietary treatment, malnutrition, and increased risk of metabolic disorders. However, our study analyzed the effect of specific meal replacement foods during FMD on blood glucose in patients with type 2 diabetes. Specific meal replacement foods were quantified and applied to fasting days. Therefore, this study not only shows the advantages of FMD in improving blood glucose in patients with type 2 diabetes but also reduces the malnutrition of patients with type 2 diabetes during FMD through the advantages of special medical food.

The result of this study showed that after the 4-month trial, FPG, 2hPG, HbA1c, BMI, waist circumference, blood pressure, and blood lipids (triglycerides, cholesterol, and low-density lipoprotein) in the test group were lower than those in the control group. The density lipoprotein level was higher than the control group, whereas the FPG, 2hPG, and blood pressure in the two groups showed a steady decline, and the decline in the test group was more significant. In addition, the HbA1c in the test group was significantly lower than that of the control group. Thus, specific meal replacement intervention during FMD can safely and effectively improve blood glucose, blood lipids, and blood pressure; reduce weight and improve metabolic parameters in patients with type 2 diabetes; improve patient compliance; achieve stable and lasting blood glucose control. The relevant mechanisms for these findings may be as follows. The pathogenesis of hyperlipidemia, type 2 diabetes mellitus, and metabolic syndrome is modulated by inflammatory cells. Short-term calorie restriction can reduce the metabolism and inflammatory activity of monocytes, thereby significantly reducing the number of inflammation-related monocytes in the blood and tissues and thereby improve the metabolic parameters [9]. Furthermore, FMD induces prenatal-development gene expression in the adult pancreas. Fasting conditions reduce PKA and mTOR activity and induce Sox2 and Ngn3 expression and insulin production. The effects of the FMD are reversed by IGF-1 treatment and recapitulated by PKA and mTOR inhibition. These results indicate that a FMD promotes the reprogramming of pancreatic cells to restore insulin generation in islets from diabetics [5]. Sutton et al. conducted a 5-week intermittent fasting study on men with prediabetes. The results showed that intermittent fasting could not improve the 24 h blood glucose level and did not affect atherosclerosis, LDL-C, and HDL-C and significantly reduced insulin levels, improved insulin sensitivity and *β*-cell reactivity, and lowered blood pressure [10]. The comparison shows that the long-term fasting simulation of decreasing food and giving specific meal replacement food intervention can better benefit the regulation of blood glucose and lipid in patients with type 2 diabetes. Wei et al. conducted a 3-month FMD test on 100 general health participants from the United States. The experimental group showed decreased levels of FPG, insulin-like growth factor 1 (IGF-1), BMI, TG, TC, LDL-C, blood pressure, and C-reactive [11]. The two groups of test subjects are different, but the results of the two groups of experiments are similar, suggesting that the intervention of specific meal replacement foods during FMD in this study is more conducive to the regulation of metabolic indicators in type 2 diabetes patients.

A limitation of this study is that the effect of specific substitute food intervention on patients with type 2 diabetes during FMD was significant. However, the participants of this study were all patients with type 2 diabetes with obesity, hypertension, and high blood lipids. Whether this diet is suitable for type 2 diabetes patients with normal weight and blood pressure needs further investigation. As we use calorie restriction based on its advantages in improving type 2 diabetes, we need to pay attention to avoid potential risks such as hypoglycemia. Exploring the influence of diet therapy on patients with type 2 diabetes for different people is a long-term goal that must be adhered to in the future research.

## 5. Conclusions

Diet therapy is the most basic treatment for type 2 diabetes. This study found that intervention through specific meal replacement foods for patients with type 2 diabetes during FMD can safely and effectively improve blood glucose, blood pressure, blood lipids, and other metabolic indicators; reduce weight; improve patient compliance; and achieve stable and lasting blood glucose control.

## Figures and Tables

**Table 1:** Baseline characteristics of the participants in the study subgroups.

Item	Control group	Test group	*P*
Male (*n* (%))	24 (48%)	22 (44%)	0.424
Age (y)	49.84 ± 1.94	50.02 ± 1.76	0.628
Course of disease (y)	5.35 ± 1.12	5.37 ± 1.00	0.925
FPG (mmol/L)	7.57 ± 0.52	7.56 ± 0.56	0.956
2hPG (mmol/L)	11.80 ± 1.42	11.97 ± 1.24	0.546
HAb1c (mmol/L)	7.93 ± 0.52	7.83 ± 0.51	0.345
Waist circumference (cm)	104.32 ± 4.84	105.10 ± 5.20	0.439
BMI (kg/m^2^)	30.12 ± 1.09	30.15 ± 1.18	0.888
TG (mmol/L)	3.93 ± 0.41	3.80 ± 0.48	0.152
TC (mmol/L)	6.18 ± 0.96	6.41 ± 1.06	0.264
HDL-C (mmol/L)	1.21 ± 0.25	1.23 ± 0.40	0.752
LDL-C (mmol/L)	3.96 ± 0.66	3.83 ± 0.73	0.335
Systolic pressure (mmHg)	155.22 ± 6.21	155.28 ± 5.96	0.960
Diastolic pressure (mmHg)	90.62 ± 6.67	91.48 ± 5.13	0.472

FPG: fasting blood sugar; 2hPG: 2-hour postprandial blood glucose; HbA1c: glycosylated haemoglobin; BMI: body mass index; TG: triglyceride; TC: serum total cholesterol; HDL-C: high-density lipoprotein cholesterol; LDL-C: low-density lipoprotein cholesterol. All values are mean ± standard deviation (SD). *P* values indicated no statistically significant differences (*P* > 0.05) on between-group comparisons.

**Table 2:** Intergroup comparison of blood glucose levels before and after the tests.

Groups	FPG (mmol/L)	2hPG (mmol/L)	HbA1c (%)
	Before the test	Before the test	Before the test
	After the test	After the test	After the test
Control	7.57 ± 0.52	11.80 ± 1.42	7.93 ± 0.52
	6.27 ± 0.37	8.33 ± 0.89	7.50 ± 0.50
Test	7.56 ± 0.56	11.97 ± 1.24	7.83 ± 0.51
	5.25 ± 0.23^a^	7.02 ± 2.27^b^	6.47 ± 0.51^c^
*t* value	-16.75	-9.84	-10.23

*n* = 50 in each group. All values are mean ± standard deviation (SD). *P* value indicated statistical significance (a, b, and c: *P* ≤ 0.001; all *P* < 0.05).

**Table 3:** Intergroup comparison of the waist circumference and BMI before and after the tests.

Groups	Waist circumference (cm)	BMI (kg/m^2^)
	Before the test	Before the test
	After the test	After the test
Control	104.32 ± 4.84	30.12 ± 1.09
	98.38 ± 4.27	28.99 ± 0.99
Test	105.10 ± 5.20	30.15 ± 1.18
	90.82 ± 4.26^a^	25.04 ± 1.00^b^
*t* value	-8.87	-19.84

*n* = 50 in each group. All values are mean ± standard deviation (SD). *P* value indicated statistical significance (a and b: *P* ≤ 0.001; both *P* < 0.05).

**Table 4:** Intergroup comparison of blood pressure before and after the tests.

Groups	Systolic pressure (mmHg)	Diastolic pressure (mmHg)
	Before the test	Before the test
	After the test	After the test
Control	155.22 ± 6.21	90.62 ± 6.67
	149.08 ± 5.50	85.20 ± 6.12
Test	155.28 ± 5.96	91.48 ± 5.13
	141.10 ± 6.67^a^	80.50 ± 5.97^b^
*t* value	-6.54	-3.89

*n* = 50 in each group. All values are mean ± standard deviation (SD). *P* value indicates statistical significance (a and b: *P* ≤ 0.001, both *P* < 0.05).

**Table 5:** Intergroup comparison of blood lipids before and after the tests.

Groups	TC (mmol/L)	TG (mmol/L)	HDL-C (mmol/L)	LDL-C (mmol/L)
	Before the test	Before the test	Before the test	Before the test
	After the test	After the test	After the test	After the test
Control	6.18 ± 0.96	3.93 ± 0.41	1.21 ± 0.25	3.96 ± 0.66
	5.60 ± 0.94	3.51 ± 0.47	1.42 ± 0.27	3.38 ± 0.62
Test	6.41 ± 1.06	3.80 ± 0.48	1.23 ± 0.40	3.83 ± 0.73
	3.63 ± 0.97^a^	2.05 ± 0.54^b^	2.28 ± 0.50^c^	1.97 ± 0.49^d^
*t* value	-10.29	-14.51	10.77	-11.96

*n* = 50 in each group. All values are mean ± standard deviation (SD). *P* value indicates statistical significance (a, b, c, and d: *P* ≤ 0.001; all *P* < 0.05).

## Data Availability

The datasets used and/or analyzed during the current study are available from the corresponding author on reasonable request.

## Supplementary Materials

Supplementary Materials The study adheres to CONSORT guidelines. “See CONSORT checklist in the Supplementary Material for comprehensive CONSORT analysis.”
